# Editorial: Long-term effects of adolescent stress, sleep deprivation, or circadian disruption on mood and anxiety

**DOI:** 10.3389/fnins.2025.1633483

**Published:** 2025-07-01

**Authors:** Chelsea A. Vadnie, Sierra J. Stringfield, Marianne L. Seney, Marcos G. Frank

**Affiliations:** ^1^David O. Robbins Neuroscience Program, Department of Psychology, Ohio Wesleyan University, Delaware, OH, United States; ^2^Department of Psychiatry, Translational Neuroscience Program, University of Pittsburgh School of Medicine, Pittsburgh, PA, United States; ^3^Center for Neuroscience, University of Pittsburgh, Pittsburgh, PA, United States; ^4^Department of Neuroscience, Translational Medicine and Physiology, Elson S. Floyd College of Medicine, Washington State University Health Sciences Spokane, Spokane, WA, United States

**Keywords:** adolescence, long-term, sleep, circadian rhythm, stress, depression, anxiety

Mood and anxiety disorders are highly prevalent and often emerge during adolescence or early adulthood (GBD 2019 Mental Disorders Collaborators, 2022; Solmi et al., 2022). Sleep disruptions, circadian rhythm disturbances, and stress are experienced by adolescents and are risk factors for mood and anxiety disorders (Heim et al., 2008; Matricciani et al., 2012; Cox and Olatunji, 2016; Vadnie and McClung, 2017; Roenneberg et al., 2019; Steiger and Pawlowski, 2019; Gariepy et al., 2020; Lindholdt et al., 2021). However, additional research is needed to determine if adolescent exposure to these factors causes long-lasting effects that lead to mood and anxiety disorders. To tackle this question, this Research Topic brings together original research and reviews of preclinical and clinical work.

In the U.S., increased adolescent suicide attempts and deaths indicate that there is a critical need to elucidate and mitigate the risk factors (Hua et al., 2023). Sexual minoritized youth are at increased risk for suicide, and it is theorized that increased stress may be to blame (Meyer, 2003; di Giacomo et al., 2018). Minoritized youth also experience sleep disruptions, which can be exacerbated by stress and are associated with suicide ideation (Liu et al., 2020; Chan and Fung, 2021; Leonard et al., 2024). Furthermore, youth with blunted reward processing are more vulnerable to suicide ideation (Tsypes et al., 2019). Seah et al. investigated if victimization stress was associated with increased suicide ideation severity, and if the association was mediated by sleep disturbance. Moreover, the authors investigated if neural responsivity to social reward moderated the relationship between victimization and sleep. The authors found that higher victimization was linked to sleep disturbances, which were associated with more severe suicide ideation. The relationship between victimization and sleep disturbance was lost in youth with higher neural responses to social reward, suggesting that interventions that enhance sensitivity to social reward would be beneficial in lessening the effects of stressors on sleep and mood.

The mental health and sleep of Korean adolescents has also drawn public concern. Suicide deaths are high (~10 per 100,000) in Korean youth, and many report insufficient sleep (Yang et al., 2005; Bertuccio et al., 2024). Jung used longitudinal data from the Korean Children and Youth Panel Survey to investigate how duration of adolescents' sleep impacts life satisfaction over time (data collected yearly for 7 years, starting with 4th graders). Sleep duration decreased by ~155 min from ages 11–17. In 7th, 8th, and 9th grade, increased sleep duration positively influenced life satisfaction during the subsequent year, suggesting that interventions that improve sleep in adolescents could have lasting beneficial effects on mental health.

Dr. Suchecki's group has been studying the effects of chronic sleep restriction (CSR) during adolescence in rats using a model that causes primarily REM sleep deprivation (Machado et al., 2004). CSR during adolescence increases anxiety-like behavior in rats (da Silva Rocha-Lopes et al., 2018), and this effect persists when rats are tested as adults (Simionato et al., 2022). Here, Barreto et al. investigated the sex-specific effects of adolescent CSR. The authors replicated the effect of adolescent CSR on anxiety-like behavior in males. However, adolescent CSR had no effect on anxiety-like behavior in females but decreased depressive-like behavior in the forced swim test. CSR impaired self-care in the sucrose splash test in both sexes. Overall, adolescent CSR resulted in sex-specific effects on anxiety and depressive-like behaviors, highlighting the importance of including sex as a biological variable.

DePoy et al. also found sex-specific effects but of circadian rhythm disruption (CRD) during adolescence. DePoy et al. exposed adolescent or adult mice to 12-h (h) reversals of the light (L)/dark (D) cycle, a paradigm that has been shown to disrupt rhythms (Kim et al., 2018). Behavior testing began once the adolescent mice reached adulthood. Adolescent CRD reduced adult anxiety-like behavior, heightened adult sensitivity to cocaine reward, and had sex-dependent effects on adult cocaine self-administration. Specifically, adult females exposed to adolescent CRD reached criteria for cocaine self-administration faster and showed enhanced cue-induced reinstatement. Adult males exposed to adolescent CRD reduced extinction responding for cocaine. Furthermore, adult CRD did not impact anxiety-like or cocaine reward behavior. These results suggest that adolescence is a vulnerable period in which CRD has long-lasting, sex-specific effects on anxiety- and substance use-related behaviors.

Rather than focusing on a light cycle paradigm that robustly disrupts rhythms, Bonilla et al. exposed mice to a L/D cycle that is less disruptive to rhythms but mimics our modern lighting conditions. Adolescent mice were exposed to a 4-week light cycle disruption (LCD) with 19 h L:5 h D for 5 days per week and 12 h L:12 h D for the remaining 2 days. Mice were returned to a typical 12 h L:12 h D schedule for 5 days before behavior testing began in adulthood. Interestingly, LCD impaired novel object recognition memory and performance in an avoidance task. LCD also altered the expression of core circadian genes in the dentate gyrus and medial amygdala, suggesting these brain regions may mediate observed behavioral changes.

Mini reviews by Guindon et al. and Chai and Bian summarize the literature investigating the effects of light-at-night and sleep disruption, respectively, on mood and anxiety disorders or relevant behaviors in animal models. Guindon et al. explain why adolescents are especially vulnerable to the disruptive effects of light-at-night and discuss possible mechanisms by which light-at-night may lead to abnormal behaviors. Chai and Bian highlight clinical evidence indicating associations between sleep and mood/anxiety during development. The authors also describe preclinical literature on the effects of adolescent sleep deprivation while pointing out possible explanations for discrepancies between studies.

In summary, clinical studies in this Research Topic support that stress and/or sleep disruption during adolescence may lead to persistent effects on suicide ideation or life satisfaction. Preclinical work indicates that adolescent sleep restriction or circadian disruption causes persistent behavior changes relevant to psychiatric disorders. However, there are still many unanswered questions regarding how these disruptions affect the adolescent brain and behavior, and how we can use that knowledge to develop therapeutics.
